# Bio-Inspired Reduced TiO_2_ Nanotube Photocatalyst Modified with Polydopamine and Silk Fibroin Quantum Dots for Enhanced UV and Visible-Light Photocatalysis

**DOI:** 10.3390/ma19020358

**Published:** 2026-01-16

**Authors:** Cristina Dumitriu, Simona Popescu, Roberta Miftode, Angela Gabriela Păun, Andreea Mădălina Pandele, Andrei Kuncser, Mihaela Mîndroiu

**Affiliations:** 1 Faculty of Chemical Engineering and Biotechnologies, National University of Science and Technology Politehnica Bucharest, Splaiul Independentei 313, 060042 Bucharest, Romania; dumitriu.cristina.o@gmail.com (C.D.); simona.popescu@upb.ro (S.P.); roberta.irodia@upb.ro (R.M.); angela.olaru@upb.ro (A.G.P.); madalina.pandele@upb.ro (A.M.P.); 2 National Institute of Materials Physics, Atomistilor 405A, 077125 Magurele, Romania; andrei.kuncser@infim.ro

**Keywords:** reduced TiO_2_ nanotubes, Silk Fibroin, quantum dots, polydopamine, interfacial charge separation, photocatalyst

## Abstract

Y-branched TiO_2_ nanotubes (NTs) were produced by anodizing titanium plates derived from aerospace production leftovers and subsequently engineered to develop an enhanced TiO_2_-based photocatalytic system. The NTs were electrochemically reduced to obtain reduced TiO_2_ nanotubes (rTN) with a narrowed bandgap, followed by surface modification with polydopamine (PD) and silk fibroin-derived quantum dots (QDs) to promote enhanced UV and visible-light photocatalysis for wastewater treatment. The QDs were hydrothermally synthesized from *Bombyx mori* silk fibroin. Scanning Electron Microscopy (SEM) revealed spherical QD agglomerates encapsulated within the PD layer, while Energy Dispersive X-ray Spectroscopy (EDX) confirmed the presence of carbon and nitrogen originating from both PD and QD. The resulting rNT/PD/QD photocatalyst exhibited a significantly reduced bandgap (1.03 eV), increased Urbach energy (1.35 eV), and moderate hydrophilicity. A high double-layer capacitance (C_dl_) indicated an enlarged electrochemically active surface due to the combination of treatments. Electrochemical characterization demonstrated reduced electrical resistance, higher charge density, and lower electron–hole recombination, leading to improved interfacial charge transfer efficiency and electrochemical stability during multi-cycle cyclic voltammetry measurements. Preliminary photocatalytic tests show that the rNT/PD/QD photocatalyst achieved a degradation efficiency of 79.26% for methyl orange (MO) and 35% for tetracycline (TC).

## 1. Introduction

Various organic pollutants are being discharged by manufacturers from different industries into aquatic environments. These organic effluents are very challenging for nature to degrade or remove. Pharmaceuticals and other chemical substances in wastewater are a major concern because they can cause health and other problems. For example, some organic dyes, frequently hazardous, may infiltrate industrial effluents that are released into aquatic systems. It can result in substantial environmental contamination. Colored contaminants diminish the penetration of sunlight into the water and interfere with the photosynthetic processes of aquatic life. Consequently, mitigating or eradicating dye pollutants from industrial effluent is essential for environmental protection [1]. Moreover, another category of pollutants, antibiotics, might cause bacterial resistance, which worries scientists [2]. Even low levels of TC can lead to harmful health effects such as hepatotoxicity and genotoxicity [3]. To solve this problem, the semiconductor catalysts function to break down antibiotic pollutants via adsorption and enhanced oxidation processes [4]. The semiconductor oxide material, when irradiated by light of energy higher than its bandgap, will produce electron/hole pairs. Then, the photogenerated hole inside the valence band can directly react with the organic molecule or with water, producing OH⋅ radicals [5]. Diverse semiconductor photocatalysts, such as TiO_2_, Bi_2_O_3_, and CeO_2_, were employed for the degradation of organic pollutants [4].

Titanium dioxide is commonly used as a photocatalyst to break down hazardous chemicals and convert them into carbon dioxide and harmless compounds [6]. Titanium dioxide nanotubes are popular for their photo-electrochemical characteristics and low cost. NT has more surface area and better electronic transport capability than porous titania [5]. Unfortunately, TiO_2_ nanotubes have a broad bandgap [4] and must absorb ultraviolet light below 387.5 nm to narrow the bandgap energy domain. However, sunlight contains only 3–5% ultraviolet light, preventing them from using the plentiful visible light (42–45%). Limited carrier transport efficiency, and elevated electron–hole recombination rate also restrict the photocatalytic breakdown rate of tetracycline and other organic pollutants [4]. To reduce the bandgap for photocatalytic applications, the electronic structure of TiO_2_ has been altered through nanostructure design, doping, and decorating with narrow bandgap semiconductors to create heterostructures [7].

The reduction process applied to NT acts on decreasing the bandgap of blue/black TiO_2_ creating purposefully defect-rich heterostructures with TiO_2_ and QDs, which is a promising method for increasing photocatalytic activity [8]. Carbon quantum dots are obtained from raw materials as non-organic carbon sources (graphite, carbon nanotubes, activated carbon, candle ash) and organic carbon sources (glucose, polyethylene glycol, bagasse, watermelon peel, amino acids, citric acid, or silk fibroin) [8]. QDs have numerous intrinsic functionalized groups, including hydroxyl (–OH), and carboxyl (–COOH). In chemical processes, these functional groups attached to the main chain (or at the end of molecules) make it easier for the crosslinking of chains with each other.

These functional groups may conjugate with a wide range of polymeric, biological, and organic materials due to their hydrophilic nature, which also improves their water solubility, adsorption capabilities, and chemical reactivity [9]. The bandgap, electrical density, and chemical activity of QDs may be tuned by doping them with heteroatoms, which provides these materials with unique optical characteristics, and boosts quantum yields; it also makes the material more electrocatalytically active [10]. Water-soluble N-QDs can be synthesized using *Bombyx mori* cocoon silk as the precursor by additive-free dissolving and hydrothermal methods [7], as it is made up of natural proteins and has up to 18% nitrogen in it [11].

To link the QDs to TiO_2_, for this research we used polydopamine, using the simple immersion of material in aqueous solutions of dopamine to create an intermediate layer. Self-polymerization of the mussel-inspired dopamine results in the formation of a thin, surface-adherent film of polydopamine on nearly any solid surface.

Dopamine and some derivatives have complex/intricate redox characteristics [12]. Enediol ligands, like dopamine, can increase the visible spectrum absorption of TiO_2_ nanoparticles, which is useful for photo-electrochemical uses [13]. Also, a lot of research has been conducted in the last few years on how dopamine interacts with fluorescent QDs because these systems are very useful for making sensor assemblies. Through different reactive oxygen processes, PD could act as an electron source that can either turn off or turn on QDs [14].

This work investigates the influence of QD binding via PD on the surface of reduced TiO_2_ nanotubes (rNTs) after a one-day immersion. The primary focus of this work is the synthesis and thorough characterization of the rNT/PD/QD photocatalyst. In line with circular economy principles, the catalyst was anodized using recycled titanium (Ti) plates sourced as production leftovers from the aerospace industry, thereby minimizing the use of critical raw materials (CRMs) and reducing reliance on primary titanium with known supply chain vulnerabilities. Beyond material sustainability, this approach demonstrates that high-purity recycled aerospace-grade Ti can serve as a reliable substrate for the fabrication of architecturally complex TiO_2_ nanotube arrays with advanced photocatalytic functionality. The novelty of this work lies in coupling strategic metal recycling with surface and nanostructural engineering, enabling the development of cost-effective and environmentally benign TiO_2_-based photocatalysts suitable for environmental remediation.

Moreover, the proposed synthesis route employs inexpensive and easy-to-process raw materials, such as bioavailable *Bombyx mori* silk fibroin and dopamine, further enhancing the sustainability of the overall process. The decoration of the NT surface with quantum dots can improve the catalytic characteristics TiO_2_-based electrodes; however, an adequate concentration of these nanoparticles is essential to prevent electron–hole recombination within the TiO_2_ crystalline structure [15]. Too many QDs can introduce defects (e.g., oxygen vacancies and/or Ti^3+^ centers) into the electrical structure, leading to significant bulk charge recombination. These variables continue to be technological challenges for the well-known TiO_2_ catalyst used in the degradation of pharmaceuticals and other compounds in wastewater. Therefore, the electrochemical properties of the synthesized rNT/PD/QD catalyst were systematically analyzed and correlated with its preliminary photocatalytic performance in the degradation of representative organic pollutants, including methyl orange (MO) and tetracycline (TC). Preliminary photocatalytic degradation tests were thus performed to demonstrate the potential of the proposed catalyst, serving as a secondary validation of its photocatalytic activity rather than as the main objective of the study.

## 2. Materials and Methods

Detailed information on reagents, instrumentation, and analytical methods is presented in the Supplementary Material. In addition, a schematic diagram illustrating the preparation steps of the photocatalyst rNT/PD/QD is provided in Figure S1.

## 3. Results and Discussion

The Supplementary Material contains comprehensive characterization data for the silk fibroin QDs, including UV–Vis and fluorescence spectroscopy results, as well as TEM images and a corresponding size distribution graph. Transmission Electron Microscopy (TEM) was employed to determine the morphological characteristics and size distribution of the QDs, as presented in Figure S2. As shown in Figure S2a, the synthesized C-QDs were uniformly dispersed, all spherical, and exhibited an average particle size of 1.88 ± 0.39 nm (Figure S2b).

### 3.1. Morphological Analysis

**Figure 1:**
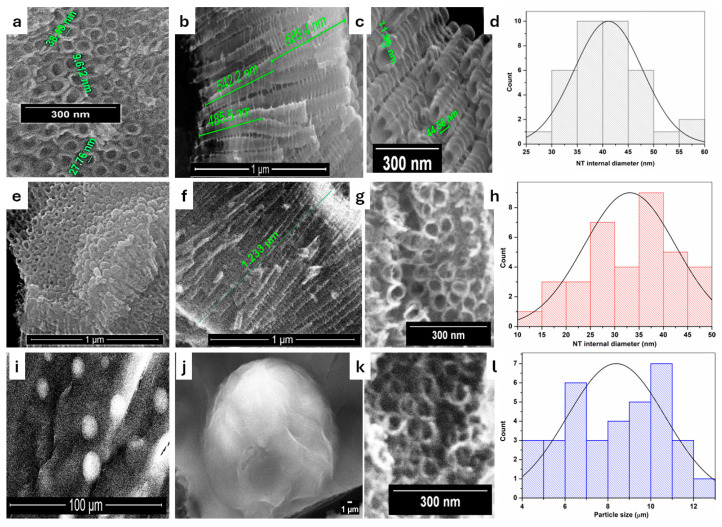
SEM images illustrating (**a**–**c**) the NT grown on Ti substrates; (**e**–**g**) reduced nanotubes rNT; (**i**–**k**) decorated nanotubes with quantum dots via polydopamine; NT internal diameters (**d**,**h**) and Particle size (**l**).

Figure 1 displays top view and side view SEM images corresponding to the NT, rNT, and rNT/PD/QD samples. The top perspective (Figure 1a) resembles the one-dimensional representation. Nanotubes have a porous architecture characterized by organized tubes resembling “honeycombs”. They have internal diameters between 25 and 60 nm, and about 10 nm for wall thickness. The internal diameter of the nanotubes was quantified from microscopic images using Image J software (version 1.54p), yielding an average value of 41.12 ± 6.69 nm (Figure 1d). From cross section image (Figure 1b), it is visible that the nanotubes are closed at the bottom part and the overall length is around 1 μm. The nanotubes’ walls have a ribbed and rough texture, as seen in Figure 1c. From Figure 1b,c, it is observable how the tubes split off, forming a “Y” shape.

After the reduction step (Figure 1e–g), the morphology of nanotubes is not affected and the tubes’ internal diameter had close values, between 10 and 50 nm. After reduction, the nanotubes exhibited a decreased internal diameter with an average value of 33.11 ± 9.40 nm (Figure 1h).The SEM results for Figure 1i sustain the successful decoration of NT with polydopamine and quantum dots, which cover the surface of NT to a large extent. It can be observed that the QDs are entrapped in the polydopamine layer as spherical clusters of few micrometers (Figure 1j,k). A quantitative evaluation was performed by measuring the diameters of 35 particles from microscopic images using Image J 1.54r, Developer: Wayne Rasband (National Institutes of Health, Bethesda, MD, USA). The analysis yielded an average diameter of 8.39 µm with a standard deviation of 2.28 µm (Figure 1l), reflecting the size distribution of the coated particles. Spatial dispersion was evaluated using nearest-neighbor distance analysis. The results show a broad distribution of inter-particle distances, suggesting moderate heterogeneity in particle spacing across the surface.

Energy-dispersive X-ray (EDX) analysis results are presented in the Supplementary Material, with representative data shown in Figure S3.

### 3.2. FT-IR Analysis

**Figure 2:**
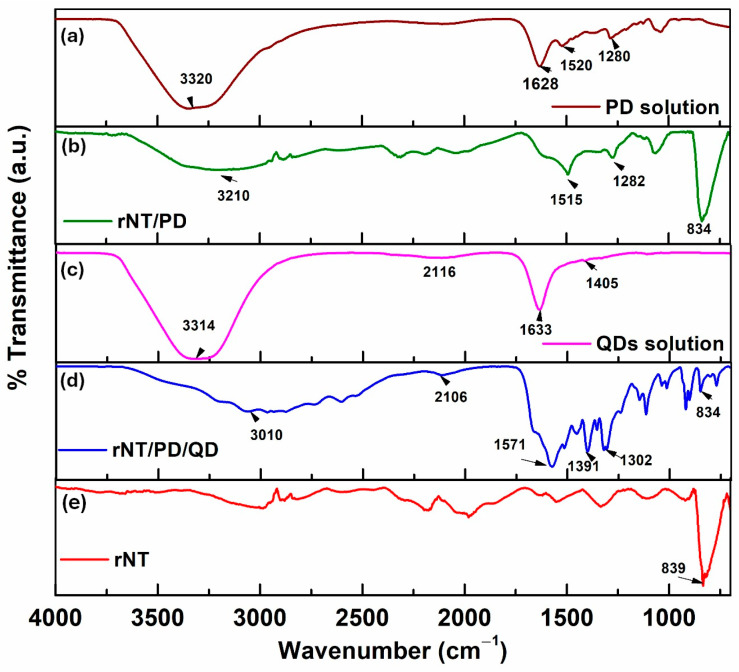
FT-IR images corresponding to the samples: (**a**) PD solution; (**b**) rNT/PD; (**c**) QDs solution; (**d**) rNT/PD/QD; (**e**) rNT.

Figure 2 presents the structural analysis of the polydopamine solution, quantum dots solution, and two Ti samples with modified surfaces with nanotubes and polydopamine, with and without quantum dots.

Polydopamine’s solution infrared absorption spectra exhibit a peak at 3320 cm^−1^, corresponding to the stretching vibrations of –OH and N-H groups (Figure 2a). Furthermore, catechol hydroxyl C=O and C–O bonds’ stretching vibrations are attributed to the peaks at 1628 cm^−1^ and 1280 cm^−1^. The peak at 1523 cm^−1^ indicates C=C bonds in PD’s indole structure [16,17,18,19,20]. The rNT/PD sample (Figure 2b) exhibits peaks at 3210, 1515, and 1282 cm^−1^, which are like those of the PD solution. This indicates that polydopamine was successfully deposited on the TiO_2_ nanotube surface.

Figure 2c shows two strong absorption peaks at 1633 cm^−1^ and 1405 cm^−1^, which correspond to the stretching vibrations of C=O and C–N. The peak at 3314 cm^−1^ corresponds to the stretching vibration of C–O in –OH and unsaturated hydroxyl groups, showing carboxyl and hydroxyl groups on the surface of the synthesized QDs [11]. The rNT/PD/QD sample shows peaks at 3010, 1571, 1391, and 1302 cm^−1^, indicating the PD and QDs were successfully attached to the electrode surface.

### 3.3. Wetting and Roughness Characteristics of Photocatalysts

The following table (Table 1) displays the data obtained from contact angle measurements used to evaluate wettability and surface free energy, which influence the interaction of the newly produced photocatalyst rNT/PD/QD with water. This interaction impacts the distribution of the polluting organic molecules on the photocatalyst surface and ultimately affects the degradation efficiency. The images recorded during contact angle measurements were presented in the Supplementary Information, Figure S5.

All examined surfaces demonstrate hydrophilic properties, with contact angle values below 60 degrees and moderate to high surface free energy (SFE). Higher hydrophilicity of the catalyst surface leads to stronger interactions with pollutant functional groups (–OH, –NH_2_). According to FTIR measurements, the presence of QDs fixed by PD on the rNT nanotubes and the inclusion of polar groups (C=O, C–N, –OH) slightly increase the hydrophilic character and SFE value of the rNT/PD/QD catalyst in comparison to rNT. Thus, the catalyst based on TiO_2_ nanotubes decorated with QDs has a strong hydrophilic property.

The roughness for NT, rNT, and rNT/PD/QD samples was determined with a roughness tester at at least five points on each sample. Average values were: 0.1176 ± 0.48 μm for NT, 0.154 ± 0.031 for rNT, and 0.239 ± 0.02 for rNT/PD/QD. According to the Wenzel model [21], decorating rNT with QDs entrapped in the PD layer as spherical clusters, as seen in the SEM images (Figure 1), increases the roughness of the nanotubes by covering the tubular layers and introducing additional micro- and nano-dimensional points, resulting in a more hydrophilic surface with more active photocatalytic reactions.

### 3.4. Optical Parameters—The Bandgap Energy and Urbach Energy

**Figure 3:**
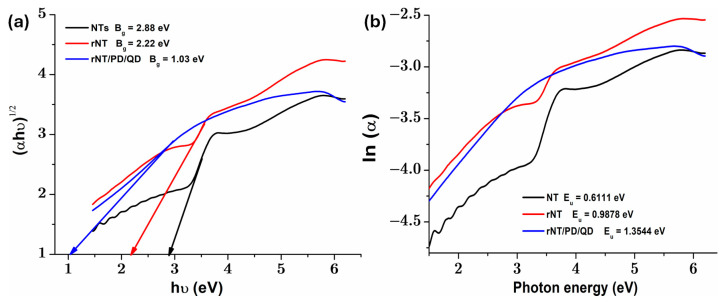
(**a**) plot of (αhν)^1/2^ versus hν and (**b**) ln(α) versus photon energy plots for obtained photocatalysts.

Semiconductor photocatalysts are widely studied for their ability to enhance solar energy, but their efficiency depends on their optical properties. TiO_2_, one of the most commonly used photocatalysts, has a relatively large bandgap of ~3.2 eV in its anatase phase, limiting its absorption primarily to the UV region of the solar spectrum. As a result, its effectiveness as a visible light absorber is significantly restricted, as noted in the literature [22]. Therefore, in photodegradation applications, it is essential to use visible-light active photocatalysts with low bandgap values. Diffuse reflectance spectra of tested samples are presented in the Supplementary Materials, Figure S6. In Figure 3a is depicted the plot of (αhν)^1/2^ versus hν, for all studied catalysts, where B_g_ is the bandgap energy, hν is the energy of the incident photon [23], and α is the absorption coefficient [24]. According to Figure 3a, when the reduction process and subsequent QD coating with polydopamine were applied, the value of the bandgap corresponding to TiO_2_ nanotubes (NT), which is approximately 2.88 eV, was lowered to 2.22 eV and 1.03 eV, respectively. Thus, fixing QDs with PD and applying the reduction treatment on the NT surface can induce surface defect bands (such as oxygen vacancies (Ov) or Ti^3+^ centers) below the conduction band, which reduce B_g_ by introducing donor states into the band [15]. This leads to a decrease in absorption in the UV region and an increase in the visible region [25,26,27].

**Figure 4:**
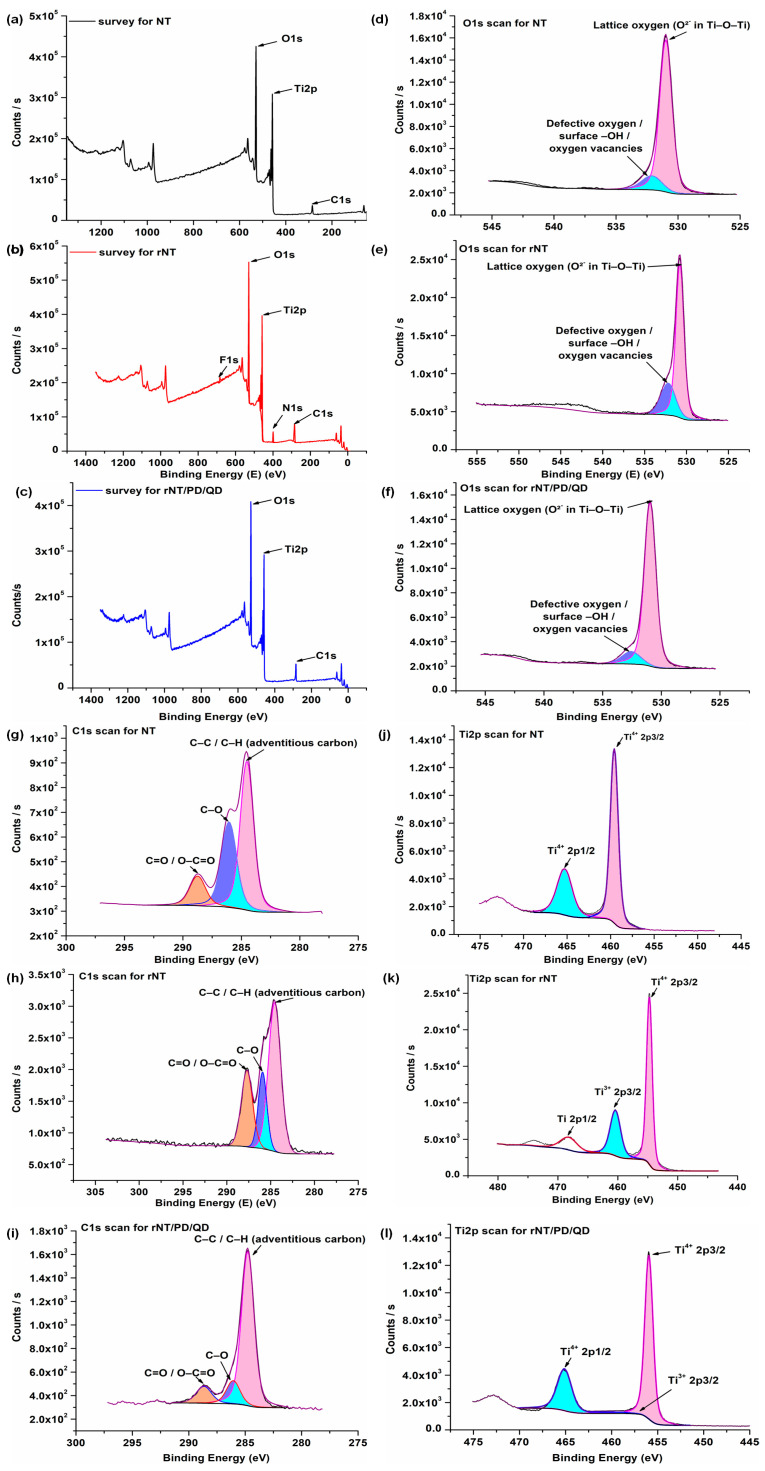
XPS results for NT (**a**–**d**), rNT (**e**–**h**), and rNT/PD/QD (**i**–**l**).

The plot in Figure 3b represents ln(α) as a function of photon energy, and the slope in the Urbach region indicates the tail of the Urbach band (E_u_), which reflects the degree of structural disorder or density of defective states in the bandgap [28]. Analysis of the optical bandgap values (Figure 3a) and Urbach energy (Figure 3b) reveals that an increase in E_u_ corresponds to a significant decrease in B_g_. This indicates that when QDs are attached to the surface of the rNT, structural defects emerge, resulting in intermediate energy levels that lead to a “narrowing” of the bandgap. The untreated NT sample shows a larger bandgap (B_g_ = 2.88 eV) and a lower Urbach energy (E_u_ = 0.61 eV), signifying a well-ordered solid structure with fewer localized states. On the other hand, the rNT/PD/QD catalyst depicts a significant decrease in B_g_ to 1.03 eV and an increase in E_u_ to 1.35 eV, indicating an increased number of defect states (Ov and/or Ti^3+^ centers) in the bandgap. As a result, the existence of these defects can trap charge, considerably reducing the energy required to excite an electron from the valence band (VB) to the conduction band (CB), as well as the rate at which electrons and holes recombine. The results corroborate the previously reported EDAX data for the rNT film (Figure 4b), indicating that the electrochemical reduction procedure increased the defect density, with the atomic ratio of oxygen to titanium approximately 2.639. The introduction of these defects into TiO_2_ structures by the electrochemical reduction approach and QDs binding has already been explored using XPS and EPR measurements in the literature [15].

### 3.5. XPS Characterization

XPS analyses were carried out on the NT, rNT, and rNT/PD/QD catalysts to investigate their surface elemental composition and chemical states. Figure 4a–c presents the survey spectra, while the corresponding high-resolution O 1s, Ti 2p, and C 1s spectra are shown in the subsequent panels. In all samples, the survey spectra reveal the presence of Ti, O, and C, confirming the TiO_2_-based nature of the nanotube structures. The carbon signal detected in the NT and rNT samples (Figure 4a,b) is attributed to adventitious carbon and residual organic species remaining on the surface after anodization and thermal treatment. In the rNT sample, additional N 1s (5.67 at.%) and F 1s (2.67 at.%) signals are observed, originating from the NH_4_F-containing ethylene glycol electrolyte used during the reduction process. For the rNT/PD/QD catalyst (Figure 4c), an increased carbon content is observed, which can be associated with the presence of the polydopamine coating and surface-functionalized quantum dots. The atomic carbon concentrations of 15.59% (NT), 24.00% (rNT), and 28.52% (rNT/PD/QD) indicate progressive surface functionalization, in good agreement with the morphological features observed in the SEM images. The oxygen content varies among the samples, reflecting changes in surface chemistry induced by reduction and functionalization processes. In particular, the modified rNT/PD/QD sample exhibits an altered O/Ti ratio, which can be related to the introduction of oxygen-containing functional groups associated with the polydopamine layer and quantum dots [29].

The high-resolution O 1s spectra of all samples (Figure 4d–f) can be deconvoluted into two main components centered at approximately 530.9 eV and 532.0 eV. The lower binding energy peak (~530.9 eV) is attributed to lattice oxygen (O^2−^) in the Ti–O–Ti framework of TiO_2_, while the higher binding energy component (~532.0 eV) is associated with surface hydroxyl groups, oxygen vacancy-related species, and adsorbed oxygen-containing species, as widely reported in the literature [30]. The relative contribution of the higher binding energy component increases for the modified samples, indicating enhanced surface hydroxylation and defect density. These findings are consistent with the contact angle measurements, which demonstrate the hydrophilic nature of the modified titanium-based samples.

Curve fitting of the high-resolution C 1s spectra of the NT, rNT, and rNT/PD/QD samples (Figure 4g–i) reveals three main components centered at binding energies of approximately 284.8 eV, 286.0 eV, and 288.0 eV. The peak at ~284.8 eV is attributed to C–C/C–H bonds arising from adventitious carbon, while the component at ~286.0 eV corresponds to C–O and C–N species. The higher binding energy peak at ~288.0 eV is associated with carbonyl and carboxyl groups (C=O and O–C=O), in agreement with previous reports [30,31,32]. In the rNT/PD/QD sample, the relative intensity of the carbonyl-related component increases, which is consistent with the presence of quinone-type structures formed during the oxidative polymerization of dopamine, thereby confirming the successful deposition of the polydopamine layer [33].

In the high-resolution Ti 2p spectrum of the NT sample, two main peaks located at binding energies of 465.2 eV and 459.6 eV are assigned to the Ti 2p_1/2_ and Ti 2p_3/2_ components of Ti^4+^ species, respectively. These spectral features are characteristic of TiO_2_ [34] and are consistent with the Raman analysis of the NT sample, which confirms the coexistence of rutile as the dominant phase and anatase as a minor phase. The observed spin–orbit splitting of approximately 5.6 eV is in good agreement with previously reported values for TiO_2_, further confirming the presence of Ti^4+^ species [32,35].

### 3.6. Electrochemical Characterization

Electrochemical tests revealed the electrical behavior of the newly formed surfaces, completing the surface characterization.

**Figure 5:**
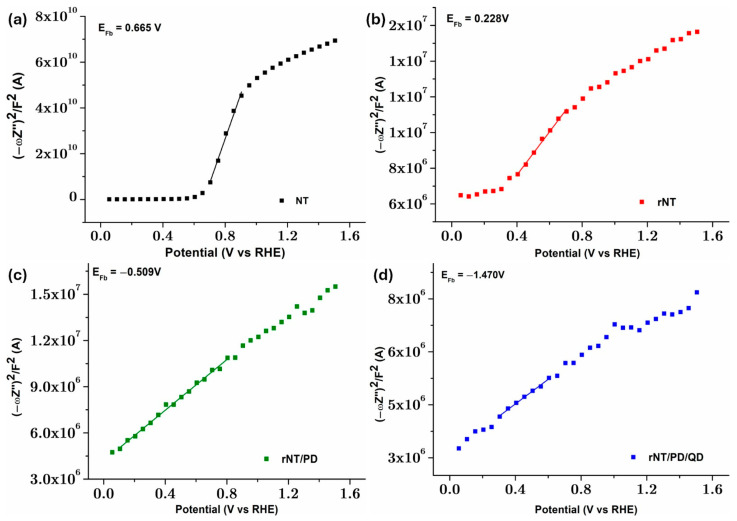
M-S plots for: (**a**) NT, (**b**) rNT, (**c**) rNT/PD, and (**d**) rNT/PB/QD electrodes.

The samples’ Mott–Schottky (M–S) plots were measured to achieve a better understanding of their interfacial charge separation efficiency (Figure 5).

The M-S curves of NT, rNT, and rNT/PB/QD exhibit positive slopes, suggesting n-type semiconductors (Figure 5) [36], signifying that the semiconductor characteristics of TiO_2_ remain unchanged by electrochemical reduction and/or PB/QD modification [15]. The flat band potential (E_fb_) and charge carrier density (N_D_) can be obtained according to Equation (1):

(1) $$\frac{1}{C^{2}}=\left(\frac{2}{q\varepsilon \varepsilon_{0}N_{D}}\right)\left(E-E_{fb}-\frac{kT}{q}\right)$$

where C is the capacity of the space charge layer, q is the elementary charge, ε_0_ is the vacuum permittivity, ε is the dielectric constant, N_D_ is the concentration of donors, E is the applied external bias, E_fb_ is the flat band potential, k is the Boltzmann’s constant, and T is the absolute temperature [25].

The E_fb_ (derived from the x-axis intercept in Figure 5a) of NT is less positively shifted from 0.67 VRHE to 0.23 VRHE following electrochemical reduction, attributed to the reducing of the surface Fermi level pinning effect [37]. After modification with PD the E_fb_ is negatively shifted to −0.51 VRHE. Bonding QDs on rNT/PD film, the E_fb_ is negatively shifted to a more negative potential of −1.47 VRHE, due to more localized defect states associated with higher Urbach energy (Figure 5b). This aspect is beneficial for facilitating the charge transfer in the electrode/electrolyte interface, resulting in enhanced photocatalytic performances [38]. The flat band potential values derived from Mott–Schottky plots are typically considered the conduction band potential (CB) for semiconductors [39].

The variations in Urbach energy (Figure 5b) are directly correlated with the changes in donor concentration (N_D_) for NT, rNT, and rNT/PD/QD. Specifically, N_D_ increases from 2.70 × 10^17^ cm^−3^ for NT to 4.32 × 10^21^ cm^−3^ for rNT and reaches 1.50 × 10^22^ cm^−3^ for rNT/PD/QD. A higher N_D_ improves the electronic conductivity, leading to improved charge separation in the electrode network [37,40]. Among the analyzed samples, rNT/PD/QD exhibits the highest interfacial charge transfer efficiency, the slowest electron–hole recombination rate, and a superior n-type conductivity compared to the NT electrode. These improvements are attributed to the presence of localized defect states introduced by the reduction treatment and the PD/QD coating. These structural changes increase light absorption at longer wavelengths, reduce the bandgap and increase the Urbach energy, and improve wetting, further optimizing catalyst performance.

**Figure 6:**
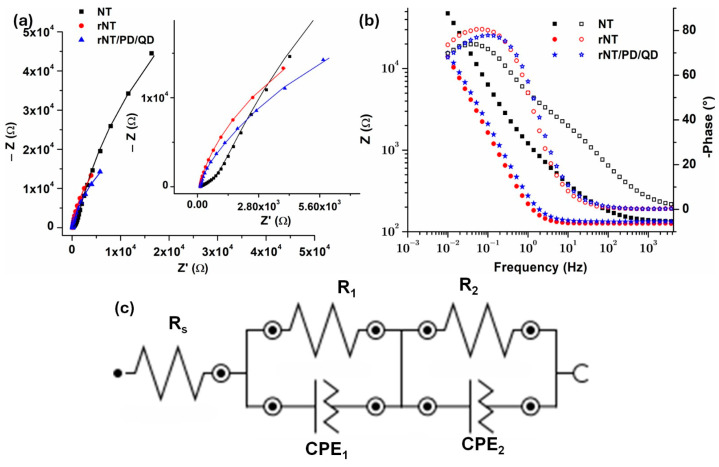
EIS results: (**a**) Nyquist Plot; (**b**) Bode plot, and (**c**) fitting circuit.

The EIS spectra recorded using Ag/AgCl 3 M KCl reference to study the interface between modified Ti surfaces (NT, rNT, and rNT/PD/QD) and 0.9% NaCl electrolyte were further compared in Figure 6. Figure 6a shows the Nyquist plot, and Figure 6b Bode diagram. To determine the most likely electrical equivalent circuit for the tested samples shown in Figure 6c, EIS data were fitted using NOVA 1.11 software. The data show that all samples exhibit considerable stability. The Nyquist impedance diagram in certain instances does not display a complete semicircle, as seen in Figure 6a. The heterogeneity present on the electrode surface induces frequency dispersion, resulting in this phenomenon [41]. The diameter of the semicircular capacitive loops exhibits a gradual rise from NT to the rNT/PD/QD sample. Capacitive loops with larger diameters provide enhanced corrosion resistance. The findings may indicate that reduction and decoration with quantum dots via dopamine might facilitate charge transfer accessibility. Also, in the Bode diagram (Figure 6b), rNT, and rNT/PD/QD samples had lower impedance modulus. All samples show comparable capacitive behavior in the lower and medium frequency domain, with almost the same slope for rNT and rNT/PD/QD.

The circuit in Figure 6c has the electrolyte solution resistance (R_s_). The solution resistance of all analyzed samples is around 100 Ω, owing to the constancy of the testing circumstances (in all cases the electrolyte was NaCl 0.9%). A resistance R_1_ in parallel with CPE_1_ may be attributed to the nanotubes layer. CPE was used instead of pure capacitance due to surface roughness, inhomogeneous reaction rates, coating thickness or composition, or non-uniform current distribution on the electrode surface. The pseudo-capacitance CPE_2_ in parallel with resistance R_2_ represents the barrier oxide layer (with or without TiO_2_ reduced and decorated with QDs), since Ti, a “valve metal,” spontaneously forms a barrier oxide layer in electrolyte solutions. The CPE is defined by Y_0_, the admittance (1/|Z|) at ω = 1 rad/s, and N, an empirical constant ranging from 0 to 1 (N = 1 indicates a pure capacitor, whereas N = 0 indicates a pure resistor). The EIS results from Table 2 show that R_2_ values assigned to the barrier oxide layer are much lower for rNT (6.7 × 10^4^ Ω) and rNT/PD/QD (5.6 × 10^4^ Ω) compared to NT (2.27 × 10^5^ Ω).

The N_2_ coefficient values (around 0.9) indicate moderate capacitor behavior of this layer. Reduction diminished the resistivity of NT, since R_1_ is lower for rNT (711.1 Ω) compared to NT (955.0 Ω). For rNT/PD/ the resistance R_1_ is the lowest at 678.42 Ω. A high donor concentration (N_D_) and an increase in electron trapping sites (such as O_v_ and/or Ti^3+^ centers), as shown by high Urbach energy values (Figure 3b), are responsible for the low resistance (R_1_) of the rNT/PD/QD layer. Increased charge transfer and enhanced catalytic efficiency are the results of these factors.

Each sample has a pseudo-capacitive nature, as shown by the N_1_ values ranging from 0.57 to 0.99. It can be concluded that reduction provides less charge transfer resistance and PD/QD conduct to markedly enhanced charge transport at the electrode/electrolyte interface.

The **χ^2^** values found varied from 0.014 to 1.05 × 10^−3^ indicating a strong concordance between the equivalent circuit and the data.

**Figure 7:**
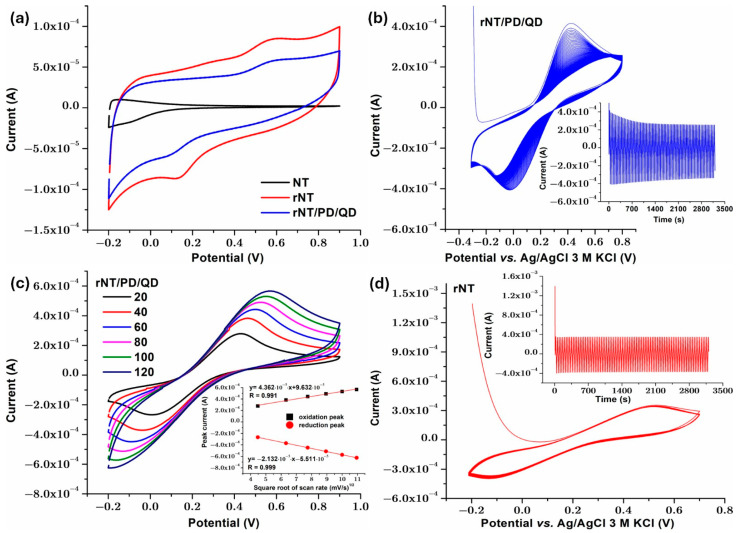
Cyclic voltammetry curves: (**a**) second cycle at 50 mV/s scan rate in NaCl 0.9% for NT, rNT, and rNT/PD/QD; (**b**) 100 cycles for rNT/PD/QD sample at 50 mV/s scan rate in NaCl 0.9% with 5 mM Fe^2+^/Fe^3+^; (**c**) at different scan rates for rNT/PD/QD in NaCl 0.9% with 5 mM Fe^2+^/Fe^3+^; (**d**) 100 cycles for rNT sample at 50 mV/s scan rate in NaCl 0.9% with 5 mM Fe^2+^/Fe^3+^.

The double-layer capacitance (C_dl_) for the analyzed samples was estimated using the CV diagrams (Figure 7a). The formula for C_dl_ is as follows [42]:

(2) $$C_{dl}=\frac{i}{\vartheta }$$

where “i” denotes the average current density measured throughout the potential sweep from 0 V to 1 V in the anodic direction, and “*ϑ*” represents the scan rate.

The presence of PD/QD film on the rNT titanium surface, with increased hydrophilicity and roughness, accelerates charge transfer at the interface and the production of electric double layers, which increases the electrochemical capacitance of nanotubular samples’ porous structures. Electrochemical capacitance is influenced by ion diffusion and charge transfer [43]. The sample rNT/PD/QD showed a high C_dl_ value (1.25 mF/cm^2^) compared to rNT (0.74 mF/cm^2^) and NT (0.04 mF/cm^2^). The increased number of active sites on the surface, caused by a high defect density and a low charge transfer resistance, may indicate improved absorption/desorption at the electrolyte solution rNT/PD/PD/Qd surface contact.

Although the rNT/PD/QD electrode exhibits lower voltametric currents compared to rNT (Figure 7a), electrochemical impedance spectroscopy reveals a reduced charge-transfer resistance, which can be mechanistically correlated with the significantly increased donor concentration (N_D_ = 1.50 × 10^22^ cm^−3^), a higher double-layer capacitance (C_dl_ = 1.25 mF/cm^−2^) and the associated changes in Urbach energy. The higher donor density enhances electronic conductivity and interfacial electron-transfer kinetics, while surface functionalization with dopamine and quantum dots modulates the electroactive surface and diffusion processes, leading to lower apparent currents but faster charge transport kinetics across the electrode–electrolyte interface.

To assess electrochemical stability, the rNT/PD/QD electrode was subjected to 100 consecutive cyclic voltammetry cycles recorded at a scan rate of 50 mV s^−1^ in 0.9% NaCl containing the Fe^2+^/Fe^3+^ redox couple. As shown in Figure 7b, the polarization curves exhibit excellent overlap over repeated cycling, indicating high electrochemical stability and good cycling durability. For comparison, the rNT electrode was evaluated under identical conditions (Figure 7c) and exhibited similarly stable voltametric behavior. The cyclic voltammogram displays a broad, featureless profile without well-defined redox peaks, indicative of a predominantly capacitive response. Although the current remains stable upon repeated cycling, the absence of distinct redox features suggests that rNT primarily enhances electronic conductivity but does not intrinsically promote specific redox processes.

Any improvement in catalytic activity, photocurrent, or pseudo-capacitive performance in nanostructures is often linked to increased surface area [44]. When photocatalyst surface area increases, organic substrate photodegradation increases. This is because surface area increases active site number [45]. To compute the active surface area of rNT/PD/QD, only a 0.5 cm^2^ geometrical area was exposed to electrolyte. Cyclic voltammetry curves were collected at several scan rates for rNT/PD/QD using 0.9% NaCl as a supporting electrolyte and equal amounts of Fe^2+^ and Fe^3+^ because theoretical studies show that redox reactions on modified electrodes exhibit surface processes at low scanning rates and diffusion control as the sweep rate rises. This implies that diffusion controls the Fe^2+^/Fe^3+^ redox process’s kinetics [46]. The findings are shown in Figure 7d. It is visible that the anodic and cathodic peak currents of the Fe^2+^/Fe^3+^ redox reaction increase linearly with the scan rate. The results shown in Figure 7d inset reveal quicker electron transport between the [Fe(CN)_6_]^3−/4−^ solution and the electrode compared to electroactive species migration, driven by concentration gradient [47,48]. The revised Randles–Sevcik equation for quasi-reversible systems [49] was used for calculation, since ΔE_p_ values are more than 59 mV:

(3) $$\mathrm{Ip}\;=\;K(\Lambda ,\alpha )(2.69\cdot 10^{5})n^{3/2}AD^{1/2}C\nu^{1/2}$$

where K(*Λ*, α) is a modified dimensionless parameter for quasi-reversible reactions n = 1 (the number of electrons), A is the active surface area of the rNT/PD/QD sample, D is the diffusion coefficient, C is the concentration in mol/cm^3^ of Fe^2+^ or Fe^3+^ (0.005 M for this study), and ν is the scan rate (0.100 V/s). The active surface area of rNT/PD/QD was found to be 2.2 cm^2^, so the prepared photocatalyst has an increased active surface area.

According to electrochemical results supported by optoelectronic and structural characterizations, a stable rNT/PD/QD catalyst with improved performance was obtained by combining electrochemical reduction treatment with QD decoration by PD. According to optoelectronic and structural characterizations, this is caused by the introduction of defects into the TiO_2_ film network. A high C_dl_ value suggests that the synergy between the treatments improved the electrochemically active surface of the rNT/PD/QD film. A high Urbach energy value means that the visible light absorption increased, and the energy bandgap was narrowed. The appropriate increase in charge density led to a decrease in electron–hole recombination, while the reduction in electrical resistance facilitated the electronic transport, according to M-S and EIS data. As a result, an improvement in the catalytic efficiency of organic compounds degradation from wastewater is expected.

### 3.7. Photocatalytic Degradation Tests

Based on the comprehensive characterization results discussed above, including the optical properties (bandgap energy and Urbach energy), as well as the electrochemical stability and electrochemical impedance spectroscopy, it can be easily distinguished that the sample that shows the highest stability and which is the most active in the presence of light is rNT/PD/QD. As a result, preliminary photocatalytic degradation studies were carried out to assess the practical performance of the selected photocatalyst in the removal of the target pollutants under irradiation.

The photocatalytic performance of the rNT/PD/QD photocatalyst was assessed by evaluating the degradation efficiency of two distinct pollutants, MO and TC, under controlled experimental conditions. The main objective was to determine if this functionalized surface could facilitate the degradation of organic contaminants and to analyze the kinetics governing the degradation processes. The photocatalytic performances of NT and rNT, as well as detailed stability tests, were not conducted in the present study. These investigations will be addressed in our future work to provide a more comprehensive understanding of the material system.

**Figure 8:**
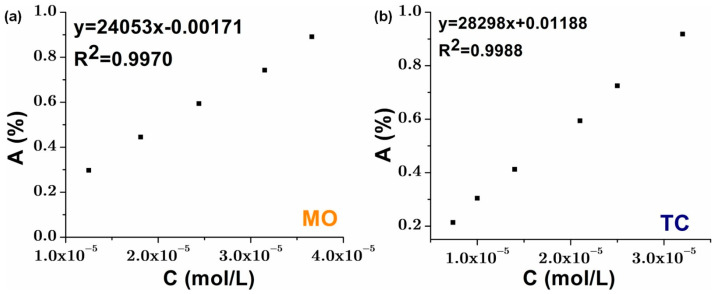
Standard calibration curves for (**a**) MO solution; (**b**) TC solution.

The calibration curves were essential for accurately determining pollutant concentrations during degradation experiments. The graphical plot with five points for MO and six for TC, ensured a reliable correlation between absorbance and concentration, following the Beer–Lambert law. A high correlation coefficient (R^2^ close to 1) confirmed measurement accuracy and method sensitivity (see Figure 8).

Any deviations from linearity could indicate molecular interactions or instrumental variations. The differences in slope and intercept between MO and TC curves reflect variations in their optical properties, which can influence degradation efficiency calculations. Ensuring precise calibration enhances the reliability of photocatalytic performance assessments.

The efficiency of the photocatalytic degradation process was assessed by monitoring the C/C_0_ ratio over time, where C represents the pollutant concentration at a given time, and C_0_ is the initial concentration.

**Figure 9:**
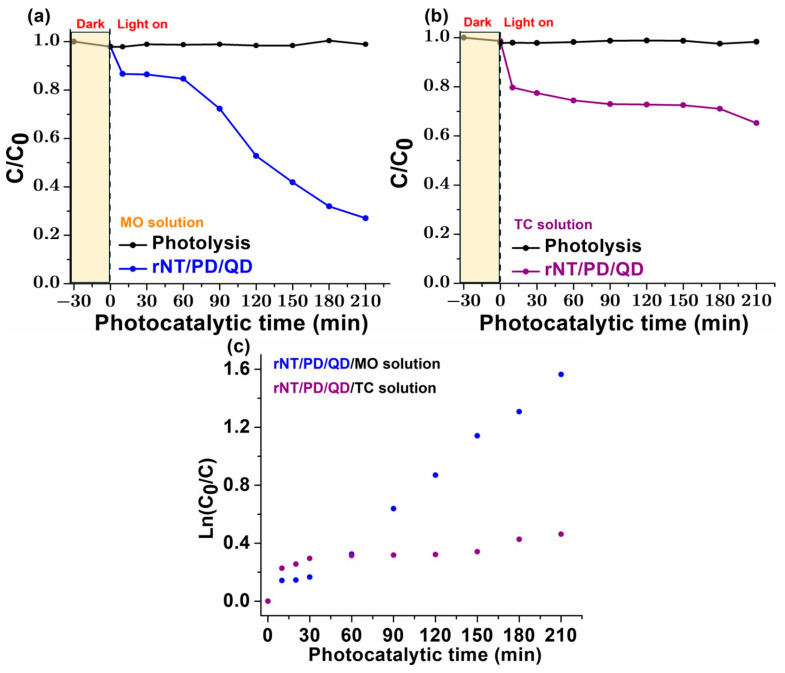
(**a**) MO photodegradation efficiency over rNT/PD/QD; (**b**) TC photodegradation efficiency over rNT/PD/QD; (**c**) First-order kinetics graph of photodegradation of MO with rNT/PD/QD.

For MO, the degradation efficiency reached an impressive 79.26%, indicating that the photocatalytic surface effectively facilitated the dye molecules degradation (Figure 9a). In contrast, the degradation efficiency for TC was lower, at 35% (Figure 9b), suggesting that the interaction between the photocatalytic surface and the antibiotic molecules was less favorable. This discrepancy could be attributed to differences in molecular structure, adsorption affinity, or photo reactivity.

Details about the degradation efficiency of the target pollutants are given in the Supplementary Material. Figure S7 gave degradation efficiency represented as a percentage under various experimental circumstances.

To gain further insight into the degradation mechanism, the reaction kinetics were analyzed using ln(C_0_/C) plots (Figure 9c). For MO, the reaction followed pseudo-first-order kinetics, with a rate constant of k = 1.613 × 10^−2^ min^−1^ and an excellent correlation coefficient (R^2^ = 0.9908). The high R^2^ value confirms that the degradation of MO is well described by the first-order kinetic model, implying that the reaction rate is directly proportional to the pollutant concentration. On the other hand kinetic data TC shows the fact that the reaction followed pseudo-first-order kinetics, with a slower rate constant of k = 3.30 × 10^−4^ min^−1^ and a good correlation coefficient (R^2^ = 0.9475) which shows its lower degradation efficiency and suggests a slower reaction rate, potentially due to differences in its molecular complexity and photocatalytic degradation pathways.

The results indicate that the rNT/PD/QD exhibits strong potential for the degradation of MO, but its effectiveness in degrading TC is limited under the tested conditions. Several factors could influence the observed differences in degradation efficiency, including the pollutant’s chemical structure, adsorption behavior on the photocatalytic surface, and the generation of reactive oxygen species. MO, an azo dye, is known to degrade efficiently under photocatalytic conditions due to its simpler aromatic structure and strong interaction with oxidizing radicals. On the other hand, TC, a more complex antibiotic, may require additional modifications to the photocatalyst or optimized reaction conditions to enhance its degradation efficiency. The photocatalytic activity of rNT/PD/QD is also corelated with MS test, which indicates the highest interfacial charge transfer efficiency and the slowest electron–hole recombination rate.

To further improve the performance of the innovative rNT/PD/QD photocatalyst for broader environmental applications, future studies could explore variations in experimental parameters such as light intensity, reaction pH, photocatalyst concentration, and exposure time. Additionally, investigating the generation of intermediate byproducts and evaluating the complete mineralization of pollutants would provide a more comprehensive understanding of the photocatalytic process. These findings contribute to the ongoing development of advanced photocatalytic materials for water treatment applications, highlighting both the strengths and limitations of the current system in degrading different classes of pollutants.

**Figure 10:**
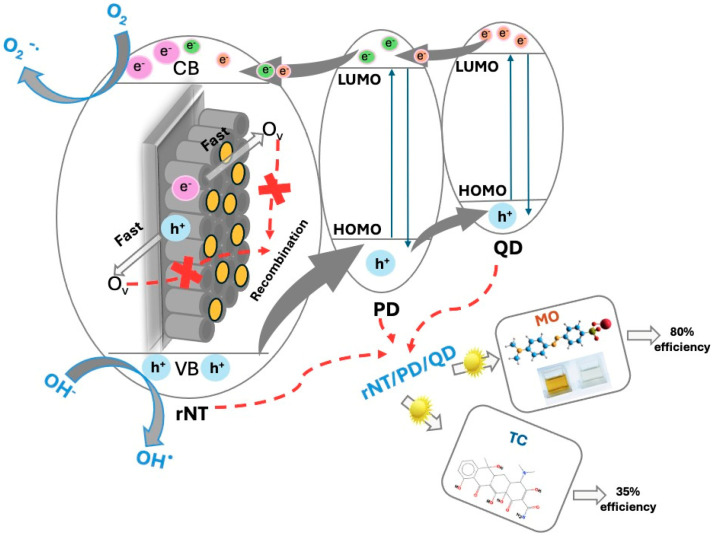
Proposed photocatalytic mechanism of the rNT/PD/QD.

A proposed photocatalytic mechanism for the degradation of the organic compounds (OC) using the rNT/PD/QD electrode is illustrated in Figure 10.

A heterojunction is established for the rNT/PD/QD electrode, in which photo-induced electrons (e^−^) are excited by the PD/QD heterojunction under UV–Vis light irradiation and transferred to the conduction band (CB) of rNT, creating vacancies (h^+^) in the valence band (VB), thereby facilitating the reactions:

rNT/PD/QD + hv → QD/PD/rNT (e^−^ + h^+^) (4)

e^−^ + O_2_ →O_2_^−^⋅ (5)

O_2_^−^⋅ + OC →.→ CO_2_ + H_2_O (6)

h^+^ + H_2_O → OH⋅ + H^+^ (7)

OH + OC →.→ CO_2_ + H_2_O (8)

The electrons excited to the conduction band of rNT, interact with oxygen adsorbed on the surface, due to high wettability (Table 1), to produce O_2_^−^⋅ [50]. The enhanced photoluminescence characteristics of QD can function as electron reservoirs, capturing photogenerated electrons and thereby enhancing charge separation [51]. Furthermore, photo-induced holes in the valence band (VB) of rNT are transferred to the HOMO (highest occupied molecular orbital) level of the PD, including the QD, for OH⋅ generation.

The defects presence in the rNT/PD/QD lattice, such as oxygen vacancies (Ov) and Ti^+3^ centers that can capture electrons can elevate the majority carrier density and create photo-induced charge traps, thereby enhancing bulk charge separation by acting as shallow donors [52]. The synergy between reduction treatment and QD decoration enhances defect density, resulting in a diminished bandgap, increased Urbach energy, raised conductivity, and reduced hole–electron recombination, which directly facilitates the photodegradation of organic compounds.

## 4. Conclusions

In this study, reduced TiO_2_ nanotubes decorated with silk fibroin-derived quantum dots via a polydopamine interlayer were successfully fabricated on titanium plates leftover from aerospace industry. The effectiveness of the surface modification was confirmed by SEM, EDX, and FT-IR analyses. SEM observations revealed that the quantum dots were encapsulated within the polydopamine layer as spherical agglomerates measuring several micrometers, while EDX analysis confirmed the presence of carbon and nitrogen species originating from both polydopamine and quantum dots.

The resulting rNT/PD/QD photocatalyst exhibited higher hydrophilicity with increased roughness, a notable reduction in bandgap to 1.03 eV, and an elevated Urbach energy (1.35 eV), signifying an augmented presence of defect states within the bandgap. These features led to enhanced interfacial charge transfer efficiency, reduced electron–hole recombination, improved n-type conductivity, and good electrochemical stability during extended multi-cycle (100 cycles) cyclic voltammetry testing.

Preliminary tests on organic compound degradation were conducted solely to assess the potential of the rNT/PD/QD photocatalyst, not as the primary objective. As a result, the photocatalyst achieved a high degradation efficiency of 79.26% for methyl orange, demonstrating effective photocatalysis driven by UV and visible light. Conversely, the degradation of tetracycline was limited to 35%, likely due to differences in molecular structure, adsorption affinity, or photochemical reactivity. Future work will focus on optimizing performance for more challenging compounds.

Overall, this work provides valuable insights into the design of bio-inspired broad-spectrum photocatalysts, focusing on the synthesis and detailed characterization of the material, while demonstrating their potential applicability in environmental remediation.

## Figures and Tables

**Table 1:** Contact angle values and surface energy values.

Sample	Contact Angle (°)			SFE (mJ/m^2^)	Molec. Attraction
	Water	EG	DMSO		
NT	33.94 + 0.25	25.09 + 1.04	20.87 + 0.81	60.20	Strong
rNT	33.15 + 0.68	17.53 + 0.25	12.86 + 0.23	60.52	Strong
rNT/PD/QD	30.28 + 0.74	14.44 + 0.15	11.03 + 0.23	62.74	Strong

**Table 2:** The optimal values of the equivalent circuit components for modified samples.

Sample	Parameter							
	R_s_ (Ω)	R_1_ (Ω)	CPE_1_		R_2_ (Ω)	CPE_2_		χ^2^
			Y_o1_ (S·s^n^)	N_1_		Y_o2_ (S·s^n^)	N_2_	
**NT**	124.7	955.0	3.5 × 10^−4^	0.570	22.7 × 10^4^	2.5 × 10^−4^	0.909	0.014
**rNT**	126.4	711.1	71.6 × 10^−4^	0.915	6.7 × 10^4^	10.9 × 10^−4^	0.985	5.3 × 10^−4^
**rNT/PD/QD**	133.6	678.42	50.0 × 10^−4^	0.990	5.42 × 10^4^	8.6 × 10^−4^	0.955	0.06361

## Data Availability

The original contributions presented in this study are included in the article/Supplementary Material. Further inquiries can be directed to the corresponding author.

## Supplementary Materials

The following supporting information can be downloaded at: https://www.mdpi.com/article/10.3390/ma19020358/s1, Figure S1: A schematic figure showing the obtaining steps for the photocatalyst: rNT/PD/QD [7,25,53,54,55]; Figure S2: (a) TEM results for QDs solution; (b) size dispersion [48,56,57,58]; Figure S3 [59]: EDX results; Figure S4: Raman spectra [11]; Figure S5: Contact angle images; Figure S6: Reflectance spectra; Figure S7: Degradation efficiency in different conditions.
