# Supplementary material for: Bio-Inspired Reduced TiO2 Nanotube Photocatalyst Modified with Polydopamine and Silk Fibroin Quantum Dots for Enhanced UV and Visible-Light Photocatalysis
Source: Materials (Basel). 2026 Jan 16;19(2):358. doi: 10.3390/ma19020358 (PMC12842894; doi:10.3390/ma19020358)
Supplement: Supplementary file 1 [file materials-19-00358-s001.zip › materials-4042502-supplementary.pdf]

materials

MDPI

Article

Bio-Inspired Reduced TiO_2 Nanotube Photocatalyst Modified with Polydopamine and Silk Fibroin Quantum Dots for Enhanced UV and Visible-Light Photocatalysis

Cristina Dumitriu [1], Simona Popescu [1], Roberta Miftode [1], Angela Gabriela Păun [1], Andreea Mădălina Pandele [1], Andrei Kuncser [2] and Mihaela Mîndroiu [1,*]

[1] Faculty of Chemical Engineering and Biotechnologies, National University of Science and Technology Politehnica Bucharest, Splaiul Independentei 313, 060042 Bucharest, Romania; dumitriu.cristina.o@gmail.com (C.D.); simona.popescu@upb.ro (S.P.); roberta.irodia@upb.ro (R.M.); angela.olaru@upb.ro (A.G.P.); madalina.pandele@upb.ro (A.M.P.)

[2] National Institute of Materials Physics, Atomistilor 405A, 077125 Magurele, Romania; andrei.kuncser@infim.ro

* Correspondence: mihaela.mindroiu@upb.ro

Experimental details

1. Preparation of photocatalyst—deposition of TiO_2 nanotube-based photocatalysts decorated with quantum dots via polydopamine

1.1. Deposition of "Y"-branched structure based on TiO_2 Nanotube Arrays

- Reagents for TiO_2 film deposition

To minimize the use of critical raw materials (CRM), recycled titanium (Ti) (Bucharest, Romania) plates 98,75% with dimensions of 2.5 cm × 3.5cm × 1 mm thickness were used as substrates. The Ti plates were sourced as production leftovers from high-precision machining processes in the aerospace industry, where excess material is generated during component fabrication. Prior to anodization, the Ti substrates were mechanically and chemically cleaned following standard protocols to remove surface contaminants and native oxides. The use of recycled aerospace-grade Ti was intentionally selected to minimize the consumption of primary titanium and to explore its suitability for the fabrication of TiO_2 nanostructures.

Other reagents included: ethylene glycol anhydrous (EG—99.8% purity), ammonium fluoride (NH4F), and sulfuric acid (H2SO4)— which were provided by Sigma Aldrich, Saint Louis, MO, USA.

- Anodization process

Two-step anodization of Ti samples was used to obtain 'Y'-branched TiO2 nanotubes. Ti plates were polished with abrasive papers of different granulometry (Carbimet, Buehler) and then cleaned in distilled water and degreased in ethanol and acetone at room temperature before anodization. The electrochemical cell was custom-made in our laboratory (Bucharest, Romania) using a Berzelius beaker as the container. The anode was a Ti electrode, while a graphite rod served as the cathode.

As electrolyte solutions, for the first anodization step a mixture of H2SO4 1 M and 0.16 M HF was used and for the second step NH4F (0.5% wt) + distilled water (2% v) + EG. Using a MATRIX MPS-7163 source, the voltage was increased from 0 to 20 V with 2 V/10 s, and then kept constant at room temperature for 2 h for each anodization step.

After the anodization process, the resulting Ti samples were ultrasonicated in distilled water for 10 seconds for the removal of the shielding layer. Then, the anodized

 https://doi.org/10.3390/ma19020358

samples were calcinated at 450 °C for 1 h. The prepared anodized Ti sample was denoted as NT.

- Reduction in TiO_2 nanotubes (NT)

The calcinated Ti samples (NT) were activated in anodization solution specific for the 2nd step at 4 V, for 10 min. The reduction process took place in the same electrolyte solution but without water: NH4F (0.25% wt) in EG solution, 40 V, for 200 seconds. The anode was represented by the anodized sample and, and a platinum electrode served as the cathode. The reduced sample was named **rNT**.

1.2. Preparation of the QDs starting from fibroin

- **Reagents for fibroin extraction**

Bombyx mori cocoons provided by a microfarm (from Stoenesti, Vâlcea county, Romania), natrium carbonate (Na_2CO_3) anhydrous, ≥ 99.5% and LiBr (≥ 99.995% trace metal base) provided by Sigma Aldrich, Saint Louis, MO, USA. Ultrapure water was obtained with a Direct-Q UV3 water purification system—Millipore(Billerica, MA, USA).

The hydro-thermal procedure outlined by Qun Wang and coworkers was used to create the QDs using silk fibroin as a precursor [1].

- Fibroin extraction

The larvae from inside the cocoons were removed and then the cocoons were sliced into little pieces. To extract sericin from the silk thread, the chopped cocoons were boiled for 30 min in a 0.02 M sodium carbonate solution. The fibroin produced was then washed to get rid of extra Na_2CO_3 and allowed to dry at room temperature. Subsequently, 1 g of dried fibroin was dissolved by adding 4 mL of LiBr 9.3 M solution, drop by drop. The procedure was carried out in an oven set at 60 °C until the color turned amber and all the fibers melted. To remove the salt, the dissolved fibroin solution was syringed into a dialysis bag (cellulose membrane, average flat width 33 mm, Merk, Darmstadt, Germany) and kept the dialysis going for 48 hours in ultrapure water. At least six water changes were required for magnetic stirring dialysis at room temperature. The estimated concentration of the fibrin solution was 7.5% by weight.

- Quantum dots synthesis

30 mL of dialyzed fibroin solution was put into a 50 mL hydro-thermal synthesis autoclave reactor with a PTFE-lined vessel (Cambridge Energy Solutions, Cambridge, UK). After that, it was placed in an electric oven that was set at 220 °C for 12 hours. The resulting solution was centrifuged (benchtop centrifuge, ROTINA 380 R, Hettich, Beverly, MA, USA) at 10,000 RPM for 30 min for the removal of impurities. Further, the clean solution was than filtered using a syringe filter PTFE (0.22 µm), as the last step to get rid of big grains. This solution was kept for later usage at 4°C and received the code QDs.

1.3. QD characterization

- Transmission Electron Microscopy (TEM)

Images of QDs have been obtained using a JEOL 2100 instrument equipped with High-resolution pole piece and Energy Dispersive X-Ray Detector (JEOL, Tokyo Japan), operated at 80 kV.

1.4. Decoration of the TiO_2 nanotubes with QDs via dopamine

- Reagents for decoration of nanotubes with QDs:

dopamine hydrochloride (98% purity), Trizma® base (NH2C(CH2OH)3), hydrochloric acid (HCl)

- Deposition of dopamine

TRIS buffer was prepared mixing Trizma® base 0.2 M solution with HCl 0.2 M solution and distilled water, as the final pH reach to 8.5. The anodized samples prepared as described above, were submerged in 5 mL of dopamine solution (2 g/L, pH 8.5) to create, at the surface of TiO_2 nanotubes, the anchors responsible for the attachment of quantum dots, in the next step. The immersion time in dopamine solution was 1 hour, at room temperature. Distilled water was used to rinse the samples.

- Decoration with QDs

The Ti modified samples were immerged into the QDs solution, at room temperature, for 24 h. The resulting sample was rinsed with distilled water to remove the unattached quantum dots. The sample name will be rNT/PD/QD.

All the preparation steps implied in the final rNT/PD/QD catalyst are synthesized in Figure S1.

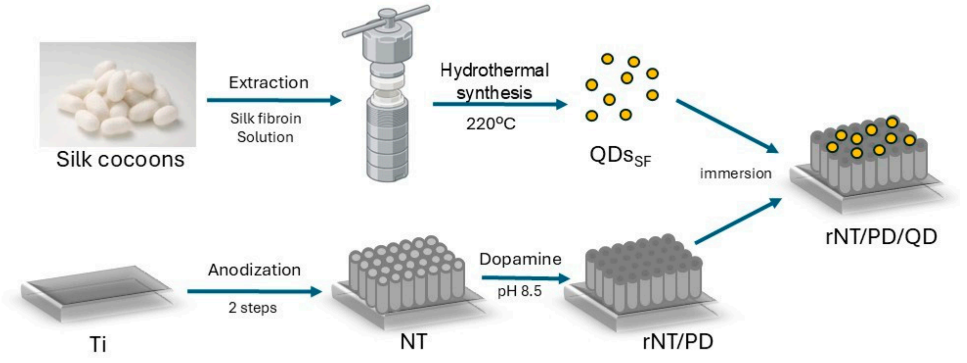

Figure S1. A schematic figure showing the obtaining steps for the photocatalyst: rNT/PD/QD.

2. *Photocatalyst characterization*

The surface morphology of the modified Ti surface was studied with an Inspect F50 high-resolution **Scanning Electron Microscope (SEM)** equipped with a Schottky Field Emission source and coupled with EDAX TEAM Basic Energy Dispersion Spectrometer (EDS) with Octane Pro Silicon Drift Detector. After being secured, the Ti samples were inserted into the microscope analysis chamber with a strip of carbon. Cross-sectional pictures were obtained by mechanically incising the anodized samples using a scalpel to fracture the nanotube layer.

X-ray photoelectron spectroscopy (XPS) was performed using a Thermo Scientific K-Alpha spectrometer (Waltham, MA, USA) that was equipped with monochromatic Al Kα X-rays (1486.6 eV) at a 90° take-off angle.

Raman spectroscopy was performed using a Horiba apparatus (Labram HR Evolution, Pailaiseau, France), a non-destructive method conducted without sample preparation. Recordings were made with an excitation wavelength of 514 nm, a 50× objective, and a 10-second acquisition period.

FT-IR spectra for the samples surface were enregistered with a Perkin-Elmer Spectrum 100, between 4000 and 600 cm−1. Four successive scans with a resolution of 4 cm−1 were registered.

The UV–VIS spectra were recorded to calculate the band gap for the Ti modified samples, using a Double Beam UV-Visible/NIR Spectrophotometer (Jasco, Tokyo, Japan) was used. Reflectance spectra were recorded between 200 and 850 nm. The band gap and Urbach energies were estimated using Tauc's equation and from the graph ln(α) = f (photon energy), respectively, according to our previous works [2,3]. The band gap was determined to highlight the contribution of quantum dots in reduction in the band gap

https://doi.org/10.3390/ma19020358

dimension. Important details regarding the incorporation of lattice defects into the NT film because of the reduction treatment and QD fixation with PD are also provided by the Urbach energy related to an extended band tail. Determinations were performed for NT, rNT, and rNT/PD/QD samples.

Surface wettability was investigated using a CAM 100 Optical Contact Angle Meter (KSV Instruments Ltd., Helsinki, Finland), employing the Sessile Drop method with three different solvents (water, EG, and DMSO). Three measurements of the contact angle value were performed at room temperature in various parts of the sample surface with three different liquids: water, ethylene glycol (reagentPlus®, ≥99%, Sigma Aldrich), and dimethyl sulfoxide (DMSO, ACS reagent, ≥99.9% Sigma Aldrich). According to the literature [4], the surface energy was computed using the Owens–Wendt model. The given values are the average values.

Surface roughness was quantified with a PCE-RT 1200 Surface Roughness Tester. A minimum of five measurements were conducted in various regions of the sample. The values provided were the mean values for each sample. The standard deviation was calculated using Excel.

- Electrochemical Characterizations

Three-electrode electrochemical cell (Metrohm, Schiedam, The Netherlands) was used for all electrochemical characterization tests. The working electrode samples were placed in a homemade plexiglass cell with an O ring to expose the same surface to the electrolyte (0.5 cm2). An Ag/AgCl in 3M KCl represented the reference electrode, a Pt rod the—counter electrode (from Metrohm, Schiedam, The Netherlands) and the working electrode was Ti with modified surface. This electrochemical cell was connected to an Autolab Potentiostat/Galvanostat PGSTAT 302N (Metrohm, Schiedam, The Netherlands). A NaCl 0.9% solution was used as the electrolyte for electrochemical characterization.

The **electrochemical impedance spectroscopy (EIS)** recorded at the open-circuit potential in the frequency range from 0.01 to 105 Hz, and the amplitude 0.01 V, were performed to investigate the interfacial charge-separation efficiencies.

A frequency of 1000 Hz was used for the **Mott–Schottky analysis**. In this experiment, the impedance of the samples was assessed at potentials varying from −0.6 to 0.85 V (relative to Ag/AgCl, 3 M KCl) in 50 mV increments. The results were obtained using a 0.01 V amplitude for the AC potential.

For **cyclic voltammetry (CV)**, the voltage was changed from −0.2 to 0.9 V (against Ag/AgCl, 3 M KCl) with a 2 mV step and 50 mV/s scan rate. CV recordings were also made different scan rates at 20 – 120 mV/s in the following electrolyte: NaCl (Sigma Aldrich, Saint Louis, MO, USA) 0.9% aqueous solution containing 5 mM potassium ferrocyanide K4[Fe(CN)6] (ACS reagent, 98.5–102.0%, Sigma Aldrich, Saint Louis, MO, USA) and 5mM potassium ferricyanide K3[Fe(CN6)] (99%, Sigma Aldirch, Saint Louis, MO, USA).

3. Photodegradation tests

Photodegradation tests were carried out in a quartz cell enclosed in a faradaic cage where outside light could not penetrate. Mercury vapor lamp used for the photodegradation process worked at 30 W and generated a white light (the lamp is fitted with filters from 200 to 600 nm wavelengths), illuminating the sample from one side of the reaction cell at around 5 cm. A D Lab MS-PA magnetic stirrer was used to control the stirring of the fluid. The solution's pH was adjusted to the desired value with NaOH and/or HCl solutions. Prior to irradiation, 10 mL of a solution with a known concentration was added in the cell, and the stirring was turned on. To reach adsorption–desorption equilibrium between the catalyst and solution, the obtained photocatalyst were submerged into solution and left in the dark for 30 min before irradiation. Following light irradiation, specific

solution volume was measured at several time points (0, 30, 60, 90, 120, 150, 180, and 210 min) for the subsequent analysis. Each experiment was conducted three times.

To evaluate the spectral changes during the photocatalytic degradation a Perkin-Elmer L950 UV–VIS/NIR spectrophotometer (Perkin Elmer, Shelton, CT, USA) was used. All the characterizations and tests were made in triplicate for maximum reproducibility, averaging them, and calculating the standard deviation with the Excel function.

The experiments involved methyl orange (MO) solution with an initial concentration of 2.44×10^{-5} mol/L and a pH of 7 as well as tetracycline (TC) solution with a concentration of 2.1×10^{-5} mol/L and a pH of 8. The photodegradation of MO was observed at 466 nm, while TC degradation was monitored at a characteristic wavelength of 356 nm. Samples were analyzed at various time points (0, 15, 30, 60, 90, 120, 150, 180, and 210 min) to study the degradation process.

The kinetic study of MO and TC photodegradation was based on a pseudo-first-order kinetic equation derived from the Langmuir–Hinshelwood model. Calibration curves were established using multiple data points to accurately quantify the photocatalytic activity. The degradation efficiency was evaluated by monitoring the concentration variation over time, expressed as C/C_0 curves, which provided insights into the reduction in the initial pollutant concentration. Furthermore, $\ln(C_0/C)$ curves were used to analyze the reaction kinetics, enabling the determination of the degradation rate and underlying reaction mechanisms (Eq. (S1)) [5]:

$$\ln\left(\frac{C_0}{C}\right) = k \times t \qquad \text{(S1)}$$

Results and discussion

1. QDs characterizations

QDs were spectroscopically characterized in another work [6] by UV–VIS, and Fluorescence spectra. The QDs exhibited strong UV–visible absorption, with a prominent peak at 220 nm attributed to a π–π^* transition. This peak suggests the presence of functional groups such as carboxyls and other electron-rich moieties that are not conjugated with the rest of the QDs structure. Additionally, a smaller absorption peak was observed at 276 nm. Under daylight, the QDs solution appeared brown, whereas it emitted green fluorescence when exposed to a 365 nm UV lamp. The fluorescence spectra of the QDs solution showed a symmetrical excitation pattern, with major peaks at 275 nm and 420 nm.

Transmission electron microscopy (TEM) was employed to determine the morphological characteristics and size distribution of the QDs, as presented in Figure S2. As shown in Figure S2a, the synthesized C-QDs were uniformly dispersed c, all of them are spherical and exhibited an average particle size of 1.88 ± 0.39 nm (Figure S2b). This aligns with the previously reported average particle size of less than 10 nm for other quantum dots [7-9].

Overall, the QDs showed nanoscale dimensions and optical behavior that align with the known characteristics of carbon dots.

https://doi.org/10.3390/ma19020358

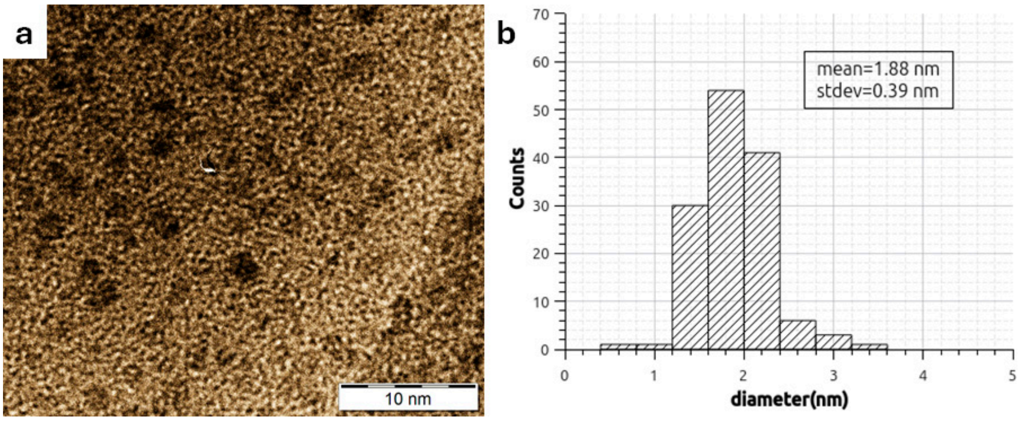

Figure S2. (a) TEM results for QDs solution; (b) size dispersion.

2. *EDX spectrum*

In order to determine the elemental composition of the NT, rNT, and rNT/PD/QD samples, the EDX spectrum was enregistered. Figure S3a indicates that the NT sample comprises the elements O and Ti, with the respective contents (at %) shown in the inset with no indication of any other impurities present. The atomic ratio of O to Ti is 2.156, which is close to 2. This signifies that the product generated after anodization is TiO2 oxide. No nitrogen or fluorine components are present in the TiO2 nanotubes post-annealing. The remaining organic molecules on the surface of TiO2 nanotubes, together with nitrogen and fluorine, are successfully eliminated during high-temperature annealing.

The rNT ratio (2.639, Figure S3b) exceeded that of NTs (2.156, Figure S4a), owing to the augmented oxygen vacancy states after the reduction process, in good agreement with literature data [10].

After deposition of polydopamine and quantum dots, the C and N elements are present from polydopamine and quantum dots. Titanium is present only in a very small amount, in good agreement with SEM images, which showed covered nanotubes by polydopamine film.

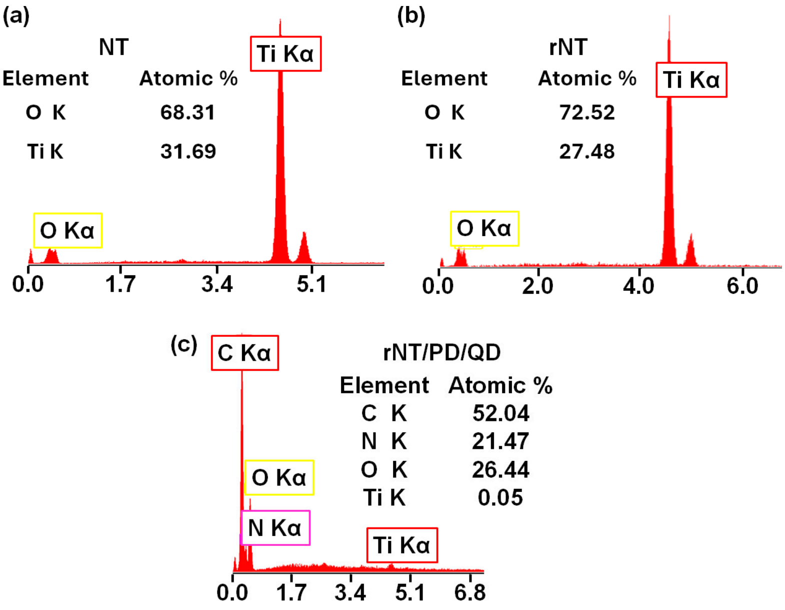

Figure S3. EDX results for: a) NT sample; b), reduced nanotubes—rNT; and c) decorated nanotubes with quantum dots via polydopamine.

https://doi.org/10.3390/ma19020358

3. Raman

The calcined NT sample was investigated by Raman spectroscopy, using commercial TiO_2 anatase and rutile powders as reference materials (Figure S4). The Raman spectrum of the commercial TiO_2 rutile powder (Figure S4a) exhibits characteristic bands at 110 cm^{-1} (B1g), 436 cm^{-1} (Eg), 590 cm^{-1} (A1g), and 690 cm^{-1} (Eg), in good agreement with the Raman-active modes (A1g + B1g + B2g + Eg) predicted by group theory and reported in the literature [11]. The Raman spectrum of the commercial TiO_2 anatase powder (Figure S4b) shows peaks at 141 cm^{-1} (Eg), 394 cm^{-1} (B1g), 511 cm^{-1} (A1g + B1g), and 635 cm^{-1} (Eg), which are consistent with literature assignments [11].

The Raman spectrum of the anodized NT sample (Figure S4c) confirms its crystalline nature and reveals the coexistence of rutile and anatase phases. Prominent rutile-related Raman bands are observed at approximately 586 cm^{-1} (A1g) and 474 cm^{-1} (Eg), the latter being slightly shifted relative to bulk rutile, which can be attributed to nanoscale effects, structural disorder, and residual stress commonly observed in anodized TiO_2 nanotube arrays. In addition, a broad composite vibrational band centered around ~250 cm^{-1} is clearly detected and can be assigned to multi-phonon scattering processes, as reported in the literature [5]. The characteristic Raman modes of anatase are also present but exhibit significantly lower intensity compared to those of rutile, indicating that anatase is present only as a minor phase in the NT sample.

https://doi.org/10.3390/ma19020358

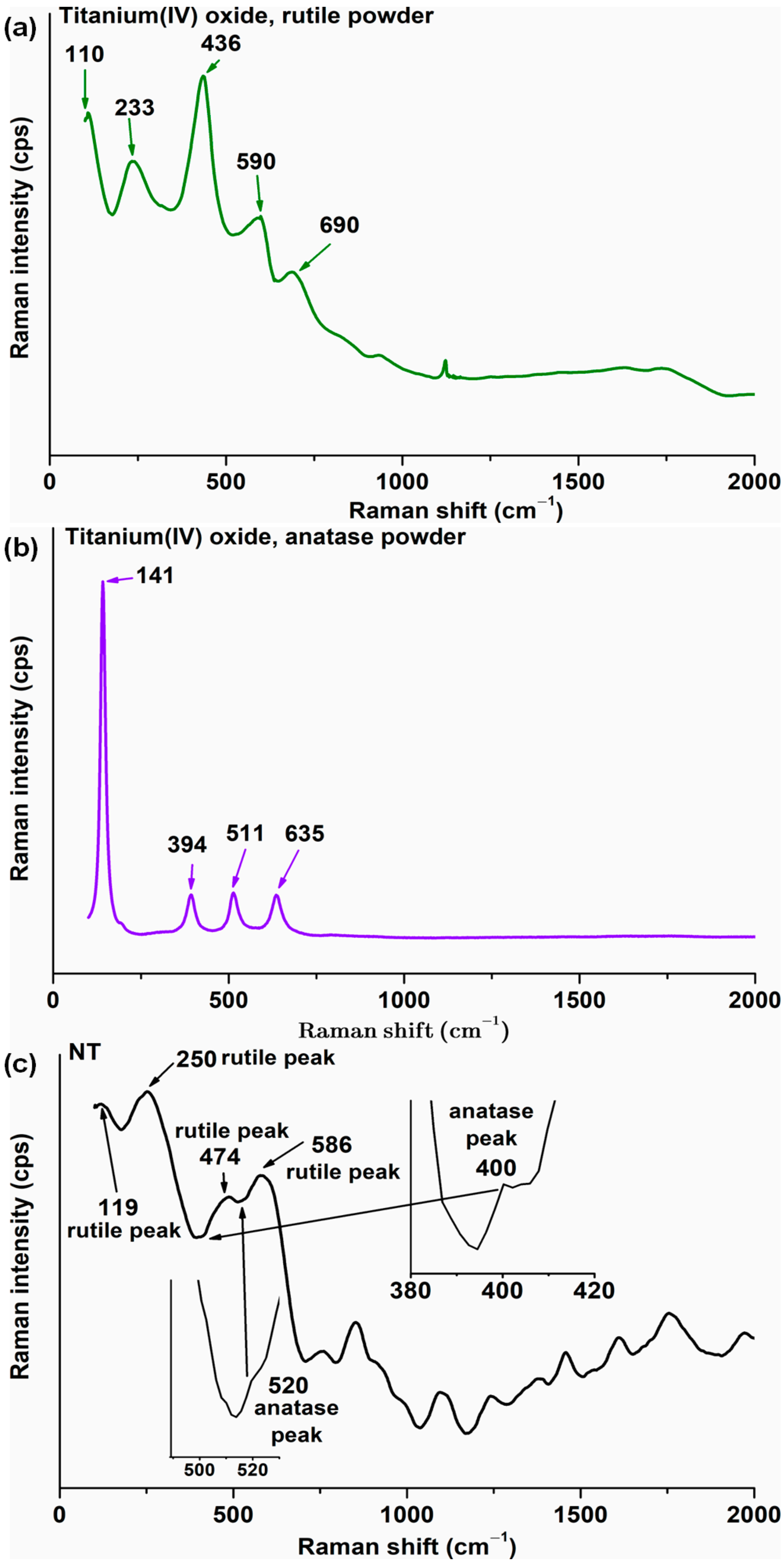

Figure S4. Raman spectra corresponding to: (a) TiO_2 rutile powder; (b) TiO_2 anatase powder; and (c) NT sample.

https://doi.org/10.3390/ma19020358

4. Contact angle images

Images recorded during measurements are presented in Figure S5.

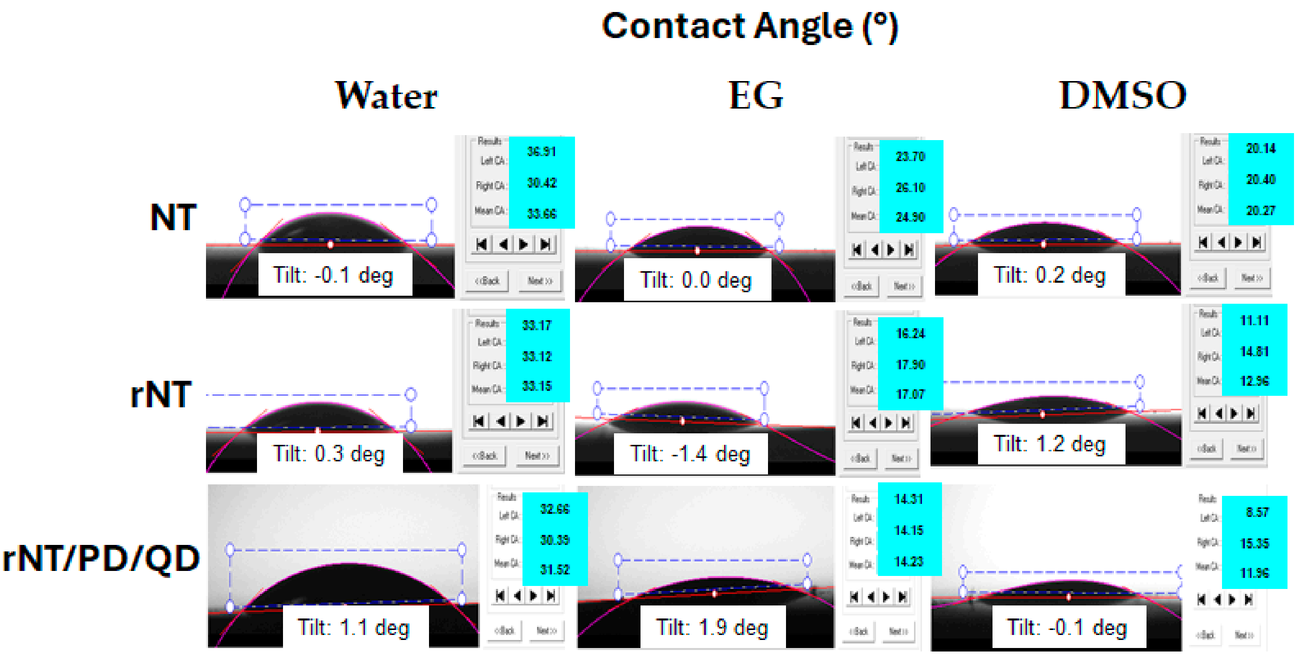

Figure S5. Contact angle images for all tested samples with water, EG, and DMSO.

5. Diffuse reflectance spectra

Recorded images are presented in Figure S6.

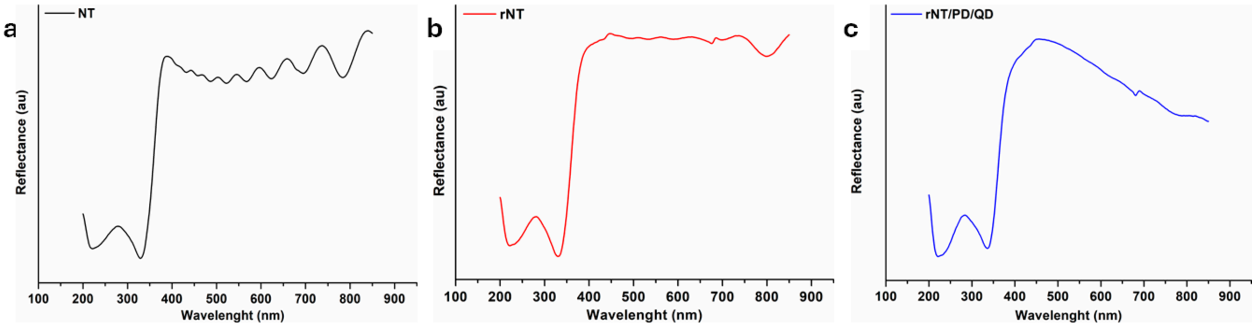

Figure S6. Reflectance spectra of tested samples a) NT, b) rNT, and c) rNT/PD/QD.

6. Degradation efficiency of the target pollutants

Figure S7 depicts the degradation efficiency of the target pollutants represented as a percentage under various experimental circumstances. All the tests were made in triplicate for maximum reproducibility, averaging them, and calculating the standard deviation with the Excel function.

Under dark conditions in both solutions, the degradation efficiency is ~2%, indicating that both pollutants are removed mostly by weak adsorption on the catalyst surface, without considerable chemical destruction. Photolysis without a catalyst for both cases yields a minimal degradation efficiency (<1%), indicating the pollutant's high photostability, and the inadequacy of direct light irradiation for efficient degradation.

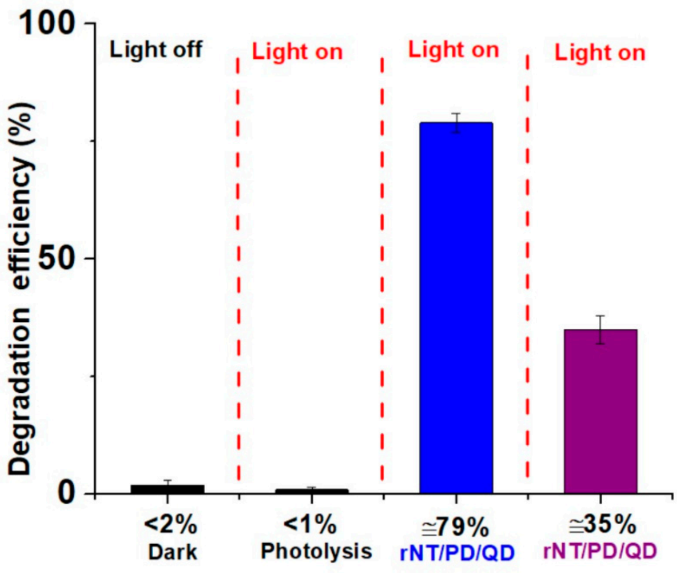

Figure S7. Degradation efficiency in different conditions.

References

1. Wang, Q.; Cai, J.; Biesold-McGee, G.V.; Huang, J.; Ng, Y.H.; Sun, H.; Wang, J.; Lai, Y.; Lin, Z. Silk fibroin-derived nitrogen-doped carbon quantum dots anchored on TiO2 nanotube arrays for heterogeneous photocatalytic degradation and water splitting. *Nano Energy* **2020**, *78*, 105313. https://doi.org/10.1016/j.nanoen.2020.105313.
2. Mîndroiu, V.M.; Stoian, A.B.; Irodia, R.; Trușcă, R.; Vasile, E. Titanium Dioxide Thin Films Produced on FTO Substrate Using the Sol–Gel Process: The Effect of the Dispersant on Optical, Surface and Electrochemical Features. *Materials* **2023**, *16*, 3147. https://doi.org/10.3390/ma16083147.
3. Irodia, R.; Ungureanu, C.; Sătulu, V.; Mîndroiu, V.M. Photocatalyst Based on Nanostructured TiO2 with Improved Photocatalytic and Antibacterial Properties. *Materials* **2023**, *16*. https://doi.org/10.3390/ma16247509.
4. Dumitriu, C.; Ungureanu, C.; Popescu, S.; Tofan, V.; Popescu, M.; Pirvu, C. Ti surface modification with a natural antioxidant and antimicrobial agent. *Surface and Coatings Technology* **2015**, *276*, 175-185. https://doi.org/10.1016/j.surfcoat.2015.06.063.
5. Challagulla, S.; Tarafder, K.; Ganesan, R.; Roy, S. Structure sensitive photocatalytic reduction of nitroarenes over TiO (2). *Scientific reports* **2017**, *7*, 8783. https://doi.org/10.1038/s41598-017-08599-2.
6. Pãun, A.G.; Popescu, S.; Ungureanu, A.I.; Trusca, R.; Popp, A.; Dumitriu, C.; Buica, G.-O. Anti-Tissue-Transglutaminase IgA Antibodies Presence Determination Using Electrochemical Square Wave Voltammetry and Modified Electrodes Based on Polypyrrole and Quantum Dots. *Biosensors* **2025**, *15*. https://doi.org/10.3390/bios15010042.
7. Elugoke, S.E.; Fayemi, O.E.; Adekunle, A.S.; Ganesh, P.-S.; Kim, S.-Y.; Ebenso, E.E. Sensitive and selective neurotransmitter epinephrine detection at a carbon quantum dots/copper oxide nanocomposite. *Journal of Electroanalytical Chemistry* **2023**, *929*, 117120. https://doi.org/10.1016/j.jelechem.2022.117120.
8. Li, H.; Zhang, Y.; Ding, J.; Wu, T.; Cai, S.; Zhang, W.; Cai, R.; Chen, C.; Yang, R. Synthesis of carbon quantum dots for application of alleviating amyloid-β mediated neurotoxicity. *Colloids and Surfaces B: Biointerfaces* **2022**, *212*, 112373. https://doi.org/10.1016/j.colsurfb.2022.112373.
9. Liu, A.-W.; Liu, J.-X.; Chen, M.-Y.; Tseng, C.-H.; Wen, Y.-J.; Chen, Y.-C.; Kuo, C.-Y. Electrospun silk fibroin nanofibers with fluorescent carbon quantum dots for efficient photo-degradation of environmental pollutants. *Journal of the Taiwan Institute of Chemical Engineers* **2025**, 106351. https://doi.org/10.1016/j.jtice.2025.106351.
10. Chen, J.; Xia, Z.; Li, H.; Li, Q.; Zhang, Y. Preparation of highly capacitive polyaniline/black TiO2 nanotubes as supercapacitor electrode by hydrogenation and electrochemical deposition. *Electrochimica Acta* **2015**, *166*, 174-182. https://doi.org/10.1016/j.electacta.2015.03.058.
11. Liu, H.; Zhang, Y.; Liu, J.H.; Hou, P.; Zhou, J.; Huang, C.Z. Preparation of nitrogen-doped carbon dots with high quantum yield from *Bombyx mori* silk for Fe(iii) ions detection. *RSC Adv.* **2017**, *7*, 50584–50590. https://doi.org/10.1039/C7RA10130A.

Disclaimer/Publisher's Note: The statements, opinions and data contained in all publications are solely those of the individual author(s) and contributor(s) and not of MDPI and/or the editor(s). MDPI and/or the editor(s) disclaim responsibility for any injury to people or property resulting from any ideas, methods, instructions or products referred to in the content.

https://doi.org/10.3390/ma19020358
